# A Nanoparticle-Based Trivalent Vaccine Targeting the Glycan Binding VP8* Domains of Rotaviruses

**DOI:** 10.3390/v13010072

**Published:** 2021-01-06

**Authors:** Ming Xia, Pengwei Huang, Xi Jiang, Ming Tan

**Affiliations:** 1 Division of Infectious Diseases, Cincinnati Children’s Hospital Medical Center, Cincinnati, OH 45229, USA; Ming.Xia@CCHMC.org (M.X.); Pengwei.huang@CCHMC.org (P.H.); 2 Department of Pediatrics, University of Cincinnati College of Medicine, Cincinnati, OH 45229, USA

**Keywords:** rotavirus, P_24_-VP8* nanoparticle, rotavirus vaccine, rotavirus VP8*, non-replicating rotavirus vaccine, norovirus P domain

## Abstract

Rotavirus causes severe gastroenteritis in children. Although vaccines are implemented, rotavirus-related diarrhea still claims ~200,000 lives annually worldwide, mainly in low-income settings, pointing to a need for improved vaccine tactics. To meet such a public health need, a P_24_-VP8* nanoparticle displaying the glycan-binding VP8* domains, the major neutralizing antigens of rotavirus, was generated as a new type of rotavirus vaccine. We reported here our development of a P_24_-VP8* nanoparticle-based trivalent vaccine. First, we established a method to produce tag-free P_24_-VP8* nanoparticles presenting the VP8*s of P[8], P[4], and P[6] rotaviruses, respectively, which are the three predominantly circulating rotavirus P types globally. This approach consists of a chemical-based protein precipitation and an ion exchange purification, which may be scaled up for large vaccine production. All three P_24_-VP8* nanoparticle types self-assembled efficiently with authentic VP8*-glycan receptor binding function. After they were mixed as a trivalent vaccine, we showed that intramuscular immunization of the vaccine elicited high IgG titers specific to the three homologous VP8* types in mice. The resulted mouse sera strongly neutralized replication of all three rotavirus P types in cell culture. Thus, the trivalent P_24_-VP8* nanoparticles are a promising vaccine candidate for parenteral use against multiple P types of predominant rotaviruses.

## 1. Introduction

Rotaviruses are non-enveloped, double-stranded RNA viruses belonging to the family Reoviridae. The virus is capsulated by three-layered capsids that are made by multiple viral proteins organizing in an icosahedral symmetry. Rotavirus is an important pathogen causing infectious acute gastroenteritis in young children, commonly resulting in dehydration and even death. Several commercial rotavirus vaccines are currently available in the international market and have helped to substantially diminish rotavirus disease burden globally [1,2]. It is noted that, however, while these live attenuated vaccines are highly effective in the industrialized world, their efficacies are apparently impaired in developing nations [3,4]. As a result, rotavirus-associated diarrhea and dehydration still cause approximately 24 million outpatient visits, 2.3 million hospitalizations, and 200,000 deaths worldwide annually [5,6]. Since most rotavirus-related diseases, morbidity, and mortality occur in the underdeveloped world, where rotavirus vaccines are most needed, a new rotavirus vaccine strategy with better effectiveness is highly demanded.

While the mechanisms behind the observed impaired effectiveness of the live rotavirus vaccines in the developing world remain elusive [7], increasing data suggest that several factors that change the intestinal conditions of vaccinated children may contribute to the reduced vaccine outcomes [8]. These factors, including malnutrition [9], concurrent uses of poliovirus and other oral vaccines [8,10], microbiota dysbiosis [11], and enterovirus infection [12], are commonly seen among children in the resource-deprived developing nations. A recent study [13] proposed a parenteral rotavirus vaccine approach to avoid these gastro-intestine-associated impacts; in other words, a nonreplicating vaccine for parenteral use may be a way to enhance the effectiveness of rotavirus vaccine in the underdeveloped countries.

To develop a nonreplicating vaccine for parenteral use, a P_24_-VP8* nanoparticle has been generated as a rotavirus vaccine candidate [14,15,16]. Each nanoparticle is composed of a 24 valent P_24_ nanoparticle core [17,18] made by 24 norovirus protruding (P) domains and 24 rotavirus VP8* antigens that are displayed on the surface of the nanoparticle. VP8* is the glycan-binding domain of rotavirus spike protein VP[4] that interacts with host glycan receptors as the first step of a rotavirus infection. Thus, VP8* serves as a key target for rotavirus vaccine development [15,19,20,21,22,23,24,25,26]. When the P-VP8* proteins are expressed using the bacterial (*Escherichia coli,* BL21 strain) system, the P_24_-VP8* nanoparticles assemble spontaneously [15]. The nanoparticles are easily manufactured at high yields with high stability [27]. They are highly immunogenic in mice [15] and pigs [16] towards the displayed VP8* antigen after intramuscular immunization and protected vaccinated mice and pigs from rotavirus challenge [15,16]. Importantly, the non-replicating P_24_-VP8* nanoparticle for parenteral immunization may also avoid the noted intussusception risk that is associated with the live rotavirus vaccines [28,29,30,31,32,33,34] and thus may deliver a better vaccine safety compared with the live vaccines. In this context, S_60_-VP8* pseudovirus nanoparticles [25,35] and rotavirus virus-like particles (VLPs) consisting of other viral proteins (VPs), including VP2, VP6, and/or VP7 [36,37], have also been generated and studied.

Rotaviruses are classified into P and G types determined by rotavirus surface proteins VP[4]/VP8* and VP7, respectively. Worldwide surveillance indicates that P[8] and P[4] are the two predominant P types in the developed world [38], accounting for ~95% of the total rotaviruses detected. While P[8] and P[4] are also predominant in developing countries, rotaviruses appear more diverse there. For example, P[6] is often found to be an important P type in Africa, contributing up to 30% of the total detected rotaviruses [39,40]. Thus, a cocktail nanoparticle vaccine containing P[8], P[4], and P[6] VP8*s representing the three major circulating P-type rotaviruses in both developed and developing worlds will most likely serve as a globally useful vaccine with broad efficacy.

The P_24_-VP8* nanoparticles of rotavirus P[8] type were produced previously by expressing the P-VP8* fusion proteins with glutathione S-transferase (GST) tags for purification purposes [15,41]. Since the 26-kDa GST tags need to be removed to allow self-formation of the P_24_-VP8* nanoparticles, this GST removal step represents a negative factor for future vaccine manufacture at a large scale. To eliminate this negative factor, we established a technology to produce and purify tag-free P-VP8* proteins and the P_24_-VP8* nanoparticles presenting VP8* antigens of P[8], P[4], and P[6] rotaviruses, respectively. This includes a selective protein precipitation by ammonium sulfate, followed by a single step of ion exchange chromatography, which may be able to scale up to large production of the P_24_-VP8* nanoparticle vaccine. The P_24_-VP8* nanoparticles were characterized biochemically, morphologically, and functionally and then mixed as a trivalent vaccine. The vaccine was studied immunologically, proving it as a promising vaccine candidate against multiple rotavirus P types circulating around the globe.

## 2. Materials and Methods

### 2.1. Plasmid Constructs

DNA sequences encoding the major functional regions of the VP8* proteins, equivalent to the amino acid sequences from L65 to L223 of VP[4], of a P[8] (strain 13851), a P[4] (strain BM5256), and a P[6] (strain 11597) rotavirus, were PCR amplified from our lab stock plasmids [22,42] and cloned into loop 2 of the modified norovirus P domain that forms P_24_ nanoparticles [15,17,18]. The resulting DNA fragments encoding the P-VP8* proteins were then subcloned into the pQE 30 vector (Novagen, Madison, WI, USA) for protein expression. The His tag was avoided by adding a stop codon before its coding sequences. The resulted P-VP8* proteins were expected to self-assemble into P_24_-VP8* nanoparticles [15].

### 2.2. Recombinant Protein Production and SDS-PAGE Analysis

The tag-free P-VP8* proteins were expressed in *Escherichia coli* (*E. coli*) (strain BL21, DE3) induced by 0.4 mM isopropyl-β-D-thiogalactopyranoside (IPTG) at room temperature (~25 °C) overnight [17,43]. For purification, clarified bacterial lyses were treated with ammonium sulfate [(NH_4_)_2_SO_4_] at 1.0 M end-concentrations for 30 min to precipitate the P-VP8* proteins. After centrifugation at 5000 rpm for 20 min with an Avanti J26XP centrifuge (Beckman Coulter, Brea, CA, USA) using a JA-17 rotor, the protein pellets were washed twice using 1 M (NH_4_)_2_SO_4_ solution in PBS. The P-VP8* protein precipitations were then solved in 20 mM Tris buffer (pH 7.5). The proteins were further purified by an anion exchange chromatography and then analyzed by gel-filtration chromatography (see below). Sodium dodecyl sulfate polyacrylamide gel electrophoresis (SDS-PAGE) was used to analyze protein quality using 10% separating gels. It was also used to quantitate proteins using serially diluted bovine serum albumin (BSA, Bio-Rad, Hercules, CA, USA) with known concentrations as standards on the same gels [15].

### 2.3. Anion Exchange and Gel Filtration Chromatography

Anion exchange chromatography was performed as described previously by Liu and coworkers using an AKTA Fast Performance Liquid Chromatography System (AKTA Pure 25L, GE Healthcare Life Sciences) via an HiPrep Q HP 16/10 column (20 mL bed volume, GE Healthcare Life Sciences, Chicago, IL, USA) at a flow rate of 2 mL/min to purify the (NH_4_)_2_SO_4_-precipitated, tag-free P-VP8* proteins. First, the column was equilibrated using 5 column volumes (CV) of 20 mM Tris-HCl buffer (pH 8.0), referred to as buffer A. Then, protein samples (5 to 9 mL) were loaded to the column, followed by washing of the column using 7 CVs of buffer A to remove the unbound proteins. The bound proteins were eluted using 7 CVs of buffer B containing 1 M NaCl in buffer A via a linear gradient elution manner (0 to 100% buffer B). The column was stripped using 7 CVs of buffer B (100% buffer B) and then equilibrated using 7 CVs of Buffer A. Relative protein concentration in the effluent was reported as A280 absorbance, while buffer composition during the elution phases was shown as a percentage of buffer B.

Gel filtration chromatography was conducted as described previously by Tan et al. [17,43,44] using the same AKTA FPLC system (see above) via a size exclusion column (Superdex 200, 10/300 GL, 25 mL bed volume, GE Healthcare Life Sciences) to analyze the size distributions of the P-VP8* proteins. The column and the elution positions of the P-VP8* proteins were calibrated using the previously made P_24_-VP8 nanoparticles (~1248 kDa) [15] and P dimers (~69 kDa) [43].

### 2.4. Electron Microscopy

Transmission electron microscopy (TEM) was utilized to analyze the formation and morphology of the P_24_-VP8* nanoparticles. The ion exchange and/or gel-filtration purified proteins were stained with 1% ammonium molybdate and inspected using an EM10 C2 microscope (Zeiss, Oberkochen, Germany) at 80 kV for magnifications between 20,000× to 30,000× [35].

### 2.5. Mouse Immunization

Rotavirus-specific-antibody-free BALB/c mice at about six weeks of age were maintained under specific pathogen-free conditions at the Division of Veterinary Services of Cincinnati Children’s Hospital Medical Center. They were randomly divided into six groups with 6 mice each (*N* = 6), and each group was immunized with one of the following immunogens: (1) a mixture of the three P_24_-VP8* nanoparticle types, representing P[8], P[4], and P[6] rotaviruses, in equal molar ratio as a trivalent vaccine at 30 µg/mouse/dose; (2) the P_24_-VP8* nanoparticles of P[8] type at 10 µg/mouse/dose; (3) the P_24_-VP8* nanoparticles of P[4] type at 10 µg/mouse/dose; (4) P_24_-VP8* nanoparticles of P[6] type at 10 µg/mouse/dose; (5) the P_24_ nanoparticles without the VP8* antigens [17,18] at 10 µg/mouse/dose as a platform control; and (6) phosphate buffer saline (PBS, pH 7.4) as vaccine diluent control. All immunogens were administered with Alum adjuvant (Thermo Scientific, Waltham, MA, USA, aluminum hydroxide, 40 mg/mL) at 25 μL/dose with 1:1 mixed with antigens at 20 μg/mouse/dose, as suggested by the manufacturer, leading to an end aluminum hydroxide dose of 1.0 mg/dose/mouse. Immunogens in 50 μL volumes were administered intramuscularly in the thigh muscle three times at 2-week intervals. Blood samples were taken 2 weeks after the final immunization through the heart puncture approach for sera samples preparations [15].

### 2.6. Enzyme Immunoassays (EIAs)

EIA was utilized to define VP8*-specific antibody titers [35]. Briefly, the previously purified GST-VP8* proteins of P[8], P[4], and P[6] rotaviruses [22,42] were coated on 96-well plates at 1 μg/mL. After blocking with 5% (*w*/*v*) skim milk, plates were incubated with mouse sera at serial 2× dilutions. Bound antibodies were measured by goat-anti-mouse IgG-horse radish peroxidase (HRP) conjugate (1:5000, MP Biomedicals, Santa Ana, CA, USA). Antibody titers were described as the maximum dilutions of sera that exhibited at least cut-off signals of OD_450_ = 0.15 as described previously by Xia and coworkers [35].

### 2.7. VP8*-Histo-Blood Group Antigen (HBGA) Binding Assays

Rotavirus VP8*-HBGA interaction was measured as described previously by Huang et al. [22,42,45]. Well-characterized saliva samples containing known HBGA types from our lab stock [46] were diluted 1000× and coated on microtiter plates. The plates were blocked with 5% (*w*/*v*) skim milk and then incubated with the purified P_24_-VP8* nanoparticles (10 µg/mL). The bound nanoparticles were detected using our in-house-made guinea pig serum against norovirus VLPs at 1:5000 dilution [46]. The bound antibodies were measured by HRP-conjugated goat anti-guinea pig IgG (1:5000, ICN, Aurora, OH, USA). The binding intensities were revealed in optical density (OD) [22].

### 2.8. Fluorescence-Based Plaque Reduction Neutralization Assays

This was done as described by Liu et al. [47]. Briefly, after a treatment with trypsin (10 μg/mL in DMEM), rotaviruses of P[8] (Wa strain, G1P8), P[6] (ST-3 strain, G4P6), and P[4] (DS-1 strain, G2P[4]) types were incubated with mouse sera at given dilutions and then added to MA104 cell monolayers in 96-well plates. After the cells were cultured in DMEM without fetal bovine serum for 16 h, the plates were frozen with 80% (*v*/*v*) acetone for 10 min at −20 °C and blocked with 2% (*w*/*v*) skim milk for 1 h at 37 °C. After the plate was incubated with anti-rotavirus antiserum (1:250) for a 1-h incubation at 37 °C, the bound antibodies were measured by fluorescein isothiocyanate (FITC)-labeled goat anti-guinea pig IgG antibodies. Plates were photographed with Cytation 5 imaging reader and fluorescence-formation plaques formed by rotavirus infected cells were enumerated. In this study, 50% neutralization titers were described as the maximum dilutions of the mouse sera showing 50% reduction in fluorescence-formation plaques.

### 2.9. Statistical Analysis

Statistical differences between the two data groups in the studies of the VP8*-specific antibody response in mice and the neutralization titers of the vaccine immunized mouse sera were determined by GraphPad Prism 7.0 (GraphPad Software, Inc., San Diego, CA, USA) through an unpaired *t* test. Differences were considered non-significant (ns) for *p*-values > 0.05 (marked as “ns”), significant for *p*-values < 0.05 (marked as *), highly significant for *p*-values < 0.01 (marked as **), and extremely significant for *p*-values < 0.001 (marked as ***), and *p*-values < 0.0001 (marked as ****), respectively.

## 3. Results

### 3.1. P-VP8* Protein Production and Purification

**Figure 1:**
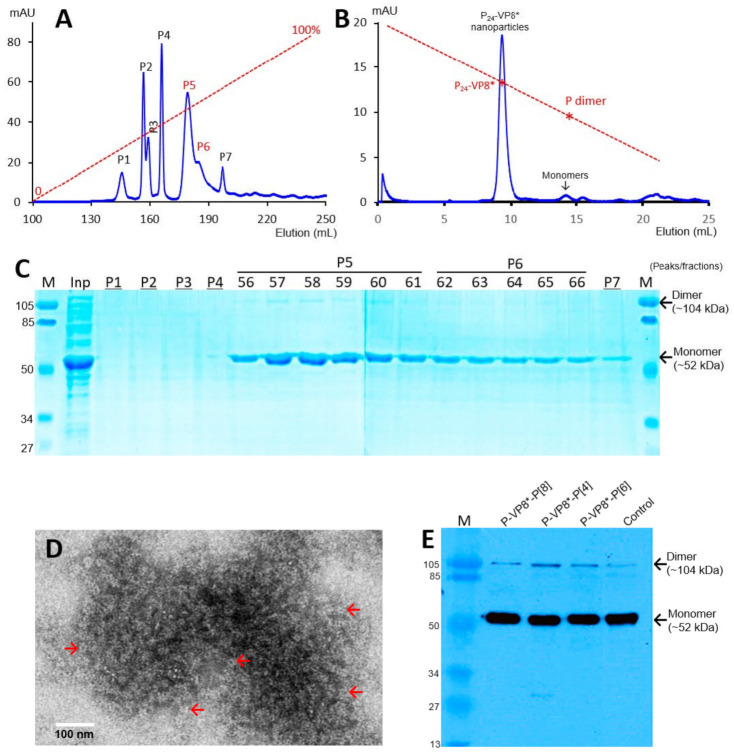
Production, purification, and characterization of the P-VP8* P[8] proteins. (**A**) A typical elution curve of an anion exchange chromatography of the (NH_4_)_2_SO_4_-precipitated P-VP8* P[8] proteins, showing elution peaks of P5 and P6 containing major P-VP8* P[8] proteins. *Y*-axis shows A280 absorbances (mAU), while *X*-axis indicates elusion volume (mL). The red dashed line shows the linear gradient increase of the elution buffer B (0–100%). (**B**) A typical elution curve of a gel-filtration chromatography of the ion exchange-purified P-VP8* protein from P5 of (**A**) through a size exclusion column. The major P_24_-VP8* nanoparticle elution peak and the minimal monomer peak are indicated. The two elution peaks were calibrated using the previously made P_24_-VP8* P[8] nanoparticles (~1.2 mDa) [15] and P dimers (69 kDa) [43] with their elution positions shown by star symbols on a red dashed line. The Y- and X-axes are the same as in (**A**). (**C**) SDS-PAGE of the major elution peaks and fractions showing the purified P-VP8* proteins. M, prestained protein standards with indicated molecular sizes in kDa; inp, the (NH_4_)_2_SO_4_-precipitated P-VP8* P[8] proteins as input proteins. (**D**) An EM micrograph of the P-VP8* (8) protein from the major peak of (**B**) showing typical P_24_-VP8 nanoparticles (arrows). (**E**) A Western blot analysis of the three ion-exchange-purified P-VP8* proteins using antibody against norovirus VLPs. The positions of both monomers and dimers are shown. Control, the previously made P-VP8* P[8] protein using the GST tag approach.

**Figure 2:**
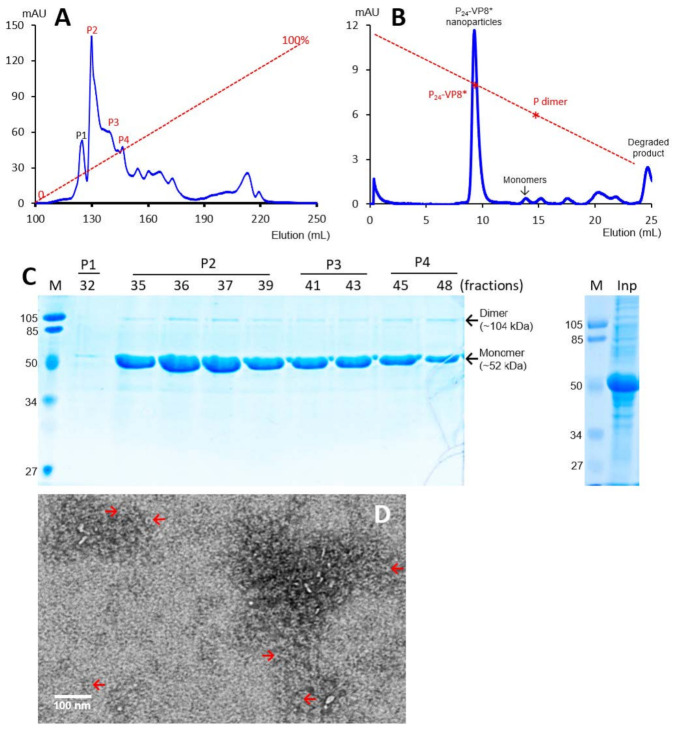
Production, purification, and characterization of the P-VP8* P[4] proteins. (**A**) A typical elution curve of an anion exchange chromatography of the (NH_4_)_2_SO_4_-precipitated P-VP8* P[4] proteins, showing elution peaks of P2, P3, and P[4] containing major P-VP8* P[4] proteins. The Y-axis shows A280 absorbances (mAU), while the X-axis indicates elusion volume (mL). The red dashed line shows the linear gradient increase of the elution buffer B (0–100%). (**B**) A typical elution curve of a gel-filtration chromatography of the ion exchange-purified P-VP8* protein from P2 of (**A**) through a size exclusion column. The major P_24_-VP8* nanoparticle elution peak and the minimal monomer peak are indicated. The two elution peaks were calibrated using the previously made P_24_-VP8* P[8] nanoparticles (~1.2 mDa) [15] and P dimers (69 kDa) [43] with their elution positions shown by star symbols on a red dashed line. The Y- and X-axes are the same as in (**A**). (**C**) SDS-PAGE showing the proteins of the major elution peaks and fractions of the purified P-VP8* P[4] proteins (left) and the (NH_4_)_2_SO_4_-precipitated proteins as input proteins (right, Inp). M, prestained protein standards with indicated molecular sizes in kDa. (**D**) An EM micrograph of the P-VP8* (4) proteins from the major peak of (**B**) showing typical P_24_-VP8 nanoparticles (arrows).

**Figure 3:**
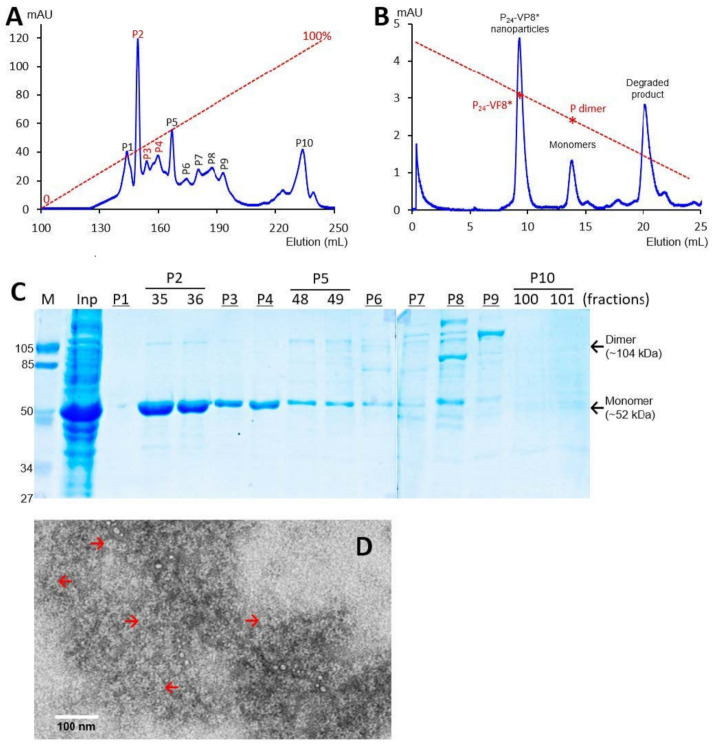
Production, purification, and characterization of the P-VP8* P[6] proteins. (**A**) A typical elution curve of an anion exchange chromatography of the (NH_4_)_2_SO_4_-precipitated P-VP8* P[6] proteins, showing elution peaks of P2, P3, and P[4] containing major P-VP8* P[6] proteins. The Y-axis shows A280 absorbances (mAU), while the X-axis indicates elusion volume (mL). The red dashed line shows the linear gradient increase of the elution buffer B (0–100%). (**B**) A typical elution curve of a gel-filtration chromatography of the ion exchange-purified P-VP8* protein from P2 of (**A**) through a size exclusion column. The major P_24_-VP8* elution peak, the minor monomer peak, and a peak representing degraded proteins with small molecular sizes are indicated. The elution peaks were calibrated using the previously made P_24_-VP8* P[8] nanoparticles (~1.2 mDa) [15] and P dimers (69 kDa) [43] with their elution positions shown by star symbols on a red dashed line. The Y- and X-axes are the same as in (**A**). (**C**) SDS-PAGE of the major elution peaks and fractions showing the purified P-VP8* P[6] proteins. M, prestained protein standards with indicated molecular sizes in kDa; inp, the (NH_4_)_2_SO_4_-precipitated proteins as input proteins. (**D**) An EM micrograph of the P-VP8*(6) proteins from the major peak of (**B**) showing typical P_24_-VP8 nanoparticles (arrows).

The recombinant P-VP8* proteins containing the VP8* antigens of the three P-type rotaviruses (P[8], P[4], and P[6]), respectively, were expressed in the *E. coli* system. The IPTG-induced bacteria were then harvested and lysed to release soluble proteins. The P-VP8* proteins in the clear fractions of the bacterial lyses were precipitated using 1 M (NH_4_)_2_SO_4_ and then further purified by anion exchange chromatography. It was noted that the three P-VP8* protein types were eluted at different salt concentrations. The P-VP8* P[8] proteins were eluted mainly in two peaks (P5 and P6, Figure 1A,C) in the latter half of the elution, corresponding to 56.8% and 62.3%, respectively, of buffer B containing 1 M NaCl. By contrast, the P-VP8* P[4] proteins were eluted mostly in three peaks (P2, P3, and P[4]) in the first half of the elution, equivalent to 29.7%, 34.6%, and 38.5% of buffer B, respectively (Figure 2A,C). Similarly, the P-VP8* P[6] proteins were eluted primarily in P2, P3, and P[4] peaks, equivalent to 25.1%, 25.9%, and 27.9% of buffer B, respectively (Figure 3A,C).

SDS PAGE showed that all three purified proteins were highly pure, reaching a purity of >90% with an expected size of about 52 kDa (Figure 1C, Figure 2C and Figure 3C). Western blot analyses using antibody against norovirus VLPs [48] confirmed that the purified proteins are P-VP8* proteins (Figure 1E). Noteworthy, small portions of P-VP8* dimers at a size of about 104 kDa were also seen (Figure 1C,E, Figure 2C and Figure 3C). Gel filtration chromatography indicated that the vast majority of the three purified P-VP8* proteins self-assembled into the P_24_-VP8* nanoparticles efficiently, as shown by a major peak (Figure 1B, Figure 2B and Figure 3B) at the position corresponding to the previously made P_24_-VP8* [8] nanoparticles using the GST tag approach [15]. Finally, EM inspection of the major elution peaks of the three gel-filtrations revealed typical morphologies of the P_24_-VP8* nanoparticles (Figure 1D, Figure 2D and Figure 3D).

### 3.2. Ligand Binding Function of the P_24_-VP8* Nanoparticles

**Figure 4:**
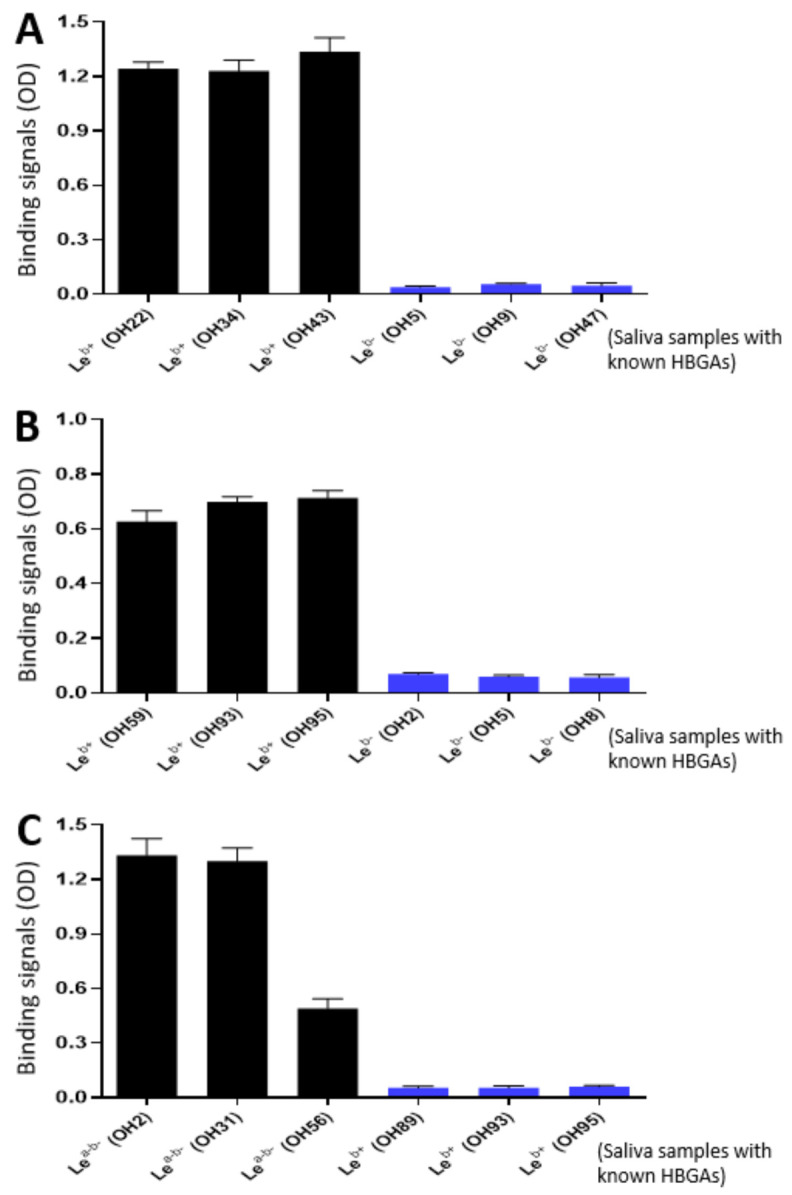
Interactions of the three P_24_-VP8* nanoparticles with glycan ligands in the well-characterized saliva samples. (**A** and **B**) The P_24_-VP8* P[8] (**A**) and the P_24_-VP8* P[4] (**B**) nanoparticles bound Le^b^ positive, but not Le^b^ negative saliva samples. (**C**) The P_24_-VP8* P[6] nanoparticles bound saliva samples with Le^a-b-^, but not Le^b+^ phenotypes. The Y-axis shows binding signals in optical density (OD), while the X-axis indicates various saliva samples with known HBGAs that are coded from OH1 to OH96 from our lab stock.

As the glycan receptor binding domains of rotaviruses, VP8*s are known to recognize specific glycan ligands. Specifically, P[8] and P[4] VP8*s bind Le^b^ antigens [22,42]. Using the saliva bank of our lab consisting of 96 well-phenotyped saliva samples and the established glycan binding methods [22,46], we found that the purified P_24_-VP8* P[8] and P_24_-VP8* P[4] nanoparticles bound Le^b^ positive, but not Le^b^ negative saliva samples (Figure 4A,B). We also screened the binding of the P_24_-VP8* P[6] nanoparticle to our 96 well-characterized saliva samples and found that it bound Le^a-b-^ saliva samples (Figure 4C). However, it remains unknown at this time what specific glycans in these saliva samples were responsible for the observed P_24_-VP8* P[6] nanoparticle-binding signals. These data strongly suggested that the VP8* antigens on the P_24_-VP8* nanoparticles retain their authentic structure and conformations, supporting the notion that the P_24_-VP8* nanoparticles are suitable to be rotavirus vaccine candidates.

### 3.3. Antibody Responses of the Trivalent P_24_-VP8* Nanoparticle Vaccine in Mice

**Figure 5:**
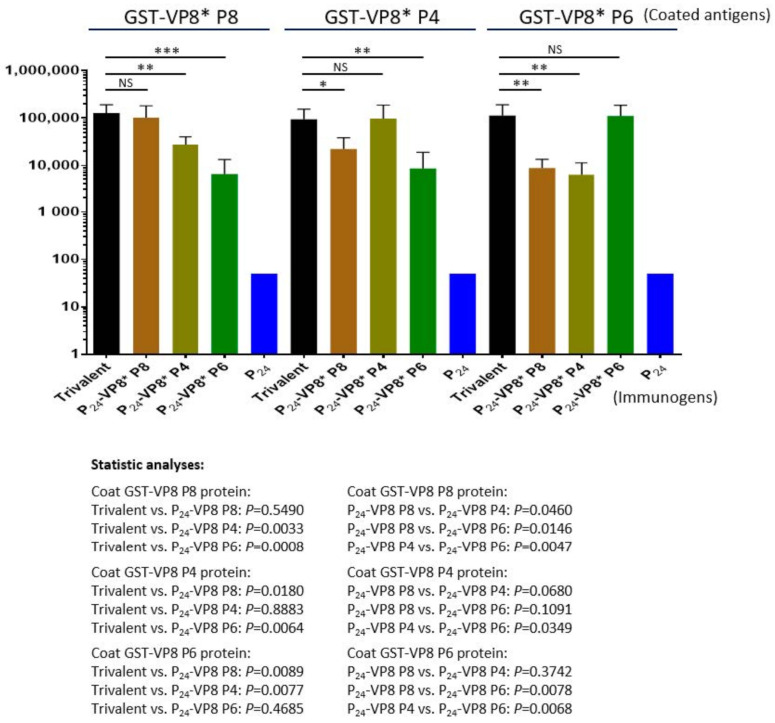
Antibody responses in mice of the trivalent P_24_-VP8* nanoparticle vaccine to the three homologous VP8* types compared with those of the three individual P_24_-VP8* nanoparticles. The Y-axis shows the VP8*-specific IgG titers, while the X-axis indicates different immunogens. Statistical differences between data groups with corresponding *P*-values are calculated and indicated below the figure. “ns”, non-significant for *p*-values > 0.05, *, significant for *p*-values < 0.05, **, highly significant for *p*-values < 0.01, ***, extremely significant for *p*-values < 0.001.

The three types of P_24_-VP8* nanoparticles were mixed at equal molar ratio as a trivalent nanoparticle vaccine to immunize mice using aluminum hydroxide as adjuvant (see Materials and Methods). Each of the three individual nanoparticles displaying the VP8* antigens of P[8], P[4], and P[6] rotaviruses, as well as the P_24_ nanoparticle without the VP8* antigen, were also immunized to mice for comparisons. The outcomes (Figure 5) showed that the trivalent vaccine induced high IgG titers (95,600 to 128,000) to VP8*s of all P[8], P[4], and P[6] types at similar IgG titers elicited by each individual homologous P_24_-VP8* nanoparticle (*Ps* > 0.05, Figure 5). However, unlike the high IgG titers to the homologous VP8*s, the sera after immunization with individual nanoparticles showed significantly lower IgG titers to the two VP8*s of heterologous P types (*Ps* < 0.05, Figure 5). It was also noted that the sera after immunization with the P[8] or P[4] nanoparticles showed higher cross reactivity to each other of the two VP8* types compared with that to the P[6] VP8*, and vice versa. These data are consistent with the fact that P[8] and P[4] rotaviruses belong to the same evolutionary lineage in the P[II] genogroup, while P[6] rotaviruses are located in another lineage of the same P[II] genogroup [49].

### 3.4. Rotavirus Neutralization by the Vaccine-Immunized Mouse Sera

**Figure 6:**
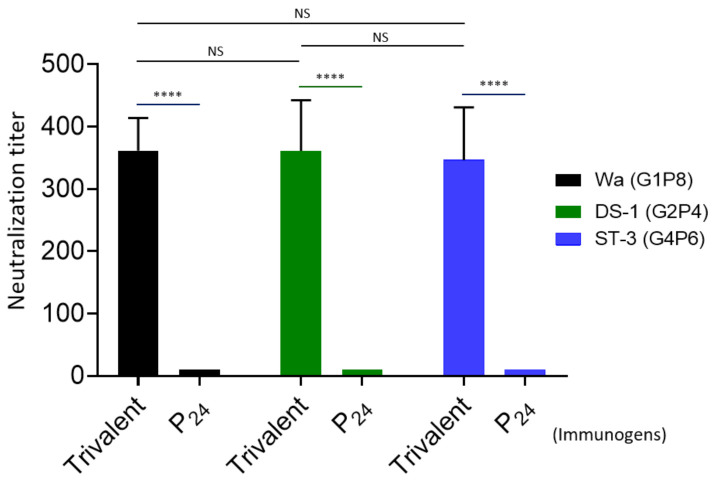
Neutralization of the P_24_-VP8* nanoparticle trivalent vaccine-immunized mouse sera against three homologous rotavirus P types. The 50% neutralization titers (Y-axis) of mouse sera after administration with the trivalent vaccine (trivalent) against a P[8] (Wa strain, G1P8, black columns), a P[4] (DS-1 strain, G2P[4], green columns), and a P[6] (ST-3, G4P6, blue columns) rotavirus in cell culture are shown and compared each other using the mouse sera after immunization with the P_24_ nanoparticles without the VP8* antigens (P_24_) as controls (X-axis). Statistical differences between data groups are indicated as “ns” for non-significant for *p*-values > 0.05, and **** for extremely significant for *p*-values < 0.0001.

The 50% neutralization titers of the mouse sera after administration with the trivalent vaccine were determined by fluorescence-based plaque reduction assays (Figure 6). Consistent with their VP8*-specific IgG titers, the trivalent vaccine-immunized mouse sera exhibited high neutralizing titers against all three rotavirus strains representing the homologous P[8] (Wa strain, G1P8), P[4] (DS-1, G2P[4]), and P[6] (ST-3, G4P6) types, reaching to titers of 362, 361, and 346, respectively. The differences between the three titers were not statistically significant (*Ps* > 0.05). These results support the notion that the P_24_-VP8* nanoparticle trivalent vaccine is a potent one against multiple major rotavirus types circulating in both developed and developing worlds.

## 4. Discussion

Rotavirus P types are determined by the surface spike protein VP[4] with its distal head VP8* interacting with host glycan receptors to initiate viral infection, making the VP8* antigens an ideal vaccine target. The P[8] rotaviruses are the most detected rotavirus P type worldwide, followed by P[4] and P[6] types. Importantly, two recent studies [50,51] indicated that VP8* plays key roles in rotavirus immune response as shown by the following: (1) ~75% of randomly isolated monoclonal antibodies (mAbs) after natural rotavirus infection are VP8*-specific; (2) ~92% of tested VP8*-directed mAbs neutralized rotavirus infection; (3) VP8*-directed mAbs protected suckling mice from human rotavirus challenge; and (4) most isolated VP8*-directed mAbs reacted with more than one VP8* type. These new data strengthen our conviction to generate P-type-based subunit rotavirus vaccines targeting the glycan receptor-binding VP8*s through the development of P_24_-VP8* (this study) and S_60_-VP8* [25,35] nanoparticle vaccines.

In this study, we generated and evaluated a trivalent P_24_-VP8* nanoparticle vaccine against the three predominant P-type rotaviruses. We first designed three types of tag-free P_24_-VP8* nanoparticles and produced them using a scalable approach that consists of simple selective precipitation by (NH_4_)_2_SO_4_, followed by a further purification through an anion exchange chromatography. After proving their nanoparticle formation with correct size, morphology, and ligand binding function, these three P_24_-VP8* nanoparticles displaying the VP8* antigens of the P[8], P[4] and P[6] rotaviruses were mixed as a trivalent vaccine. Mouse immunization study showed that the trivalent vaccine elicited high antibody responses in mice to VP8*s of all three P types and the resulted mouse sera neutralized rotavirus replication of all three rotavirus P types in cell cultures. These data strongly suggested that our trivalent P_24_-VP8* nanoparticles are a promising parenteral vaccine candidate against rotavirus-associated diseases.

The P-VP8* proteins were made previously as GST-tagged fusion proteins for purification purposes [15], and the large GST (26 kDa) tag needs to be removed to allow the free P-VP8* protein to self-assembling into the P_24_-VP8* nanoparticles. This GST removal step will impose a negative impact on future large production of the P_24_-VP8* nanoparticle vaccine. Thus, a simpler, scalable production approach is needed. We presented evidence here that tag-free P-VP8* protein can be selectively precipitated by (NH_4_)_2_SO_4_. After a further purification step by anion exchange chromatography, the P-VP8* protein reaches a high purity. Importantly, this method also increases the production yields of the P-VP8* proteins compared with those using the GST-tag method based on our lab-scale evaluation (data not shown). The tag-free P-VP8* proteins were shown to self-assemble into P_24_-VP8* nanoparticles efficiently with the expected glycan-binding function, validating the production and purification methodology for vaccine purpose.

The trivalent vaccine elicited similar high IgG titers to all P[8], P[4], and P[6] VP8*s, and the mouse sera revealed equally high neutralization titers to all the three rotavirus P types, while the single valent P_24_-VP8* nanoparticles induced significantly lower IgG titers to the two heterologous VP8* antigens. These results indicate a necessity of a cocktail or trivalent P_24_-VP8* nanoparticle vaccine for a broader efficacy. Sequence alignments of the three VP8*s show that P[8] and P[4] VP8*s share higher sequence identity (84.3%) than those between P[8]/P[4] and P[6] (59.7%/60.4%), which explains the observed discrepancies in IgG cross reactivity. Similarly, consistent with their IgG titers, the mouse sera after immunization with the trivalent vaccine showed high neutralizing titers against all three P-type rotaviruses in cell culture. Although it has not yet been done, we anticipate that the mouse sera after immunization with each single-valent P_24_-VP8* nanoparticle would show lower neutralizing titers against the two heterologous P-type rotaviruses based on their cross-reactive IgG titers. These data support the trivalent P_24_-VP8* nanoparticles being a potent vaccine with broad effectiveness. Ideally, we should also examine the cross-antigenic reactivity and cross-neutralization titers against more heterologous P types of VP8*s and rotaviruses for a comprehensive understanding of the further broad efficacy of our trivalent vaccine. However, this could not be completed at this time due to the lack of necessary resources. To reach this goal in the near future, we plan to prepare more recombinant VP8* proteins representing further rotavirus P types to evaluate the breadth of the cross antigenic reactivity of the trivalent vaccine-immunized mouse sera. We also plan to reach out to other labs for additional cell-culture-adapted rotavirus strains of other heterologous P types for future neutralization study.

The equal molar ratio of the three individual P_24_-VP8* nanoparticles representing the three VP8* types, respectively, in the current trivalent vaccine is based on the hypothesis that each monovalent antigen elicits similar immune responses by the same amount of antigens. This hypothesis has now been proved by comparing the similar IgG responses of the trivalent vaccine towards each of the three VP8* types (Figure 5). In fact, the three individual monovalent vaccines also showed similar IgG responses. On the other hand, we wished to explain that the principle of animal sera after immunization with VP8* antigens blocking interaction of rotavirus VP8*s with their glycan ligands has been well proved by our previous studies [35,47,52]. This blocking assay has been proposed as a surrogate neutralization assay in case cell-culture-based neutralization assay cannot be performed. In this study, since we have conducted cell-culture-based neutralization assays, we feel that it is not necessary to do the VP8*-glycan binding/blocking based surrogate neutralization assay at the same time.

Several VP8*-based subunit rotavirus vaccine candidates are currently under development, among which a tandem truncated VP8* with a T cell epitope, named P2-VP8 [53], has been in clinical trials, showing its safety and immunogenicity for parenteral use in humans [54]. This P2-VP8 vaccine contains only two fused VP8* copies per molecule with a small molecular size of ~20 kDa [53]. This simple molecular set up of P2-VP8* differs strikingly from our P_24_-VP8* nanoparticle, which has 24 VP8* antigens per nanoparticle and a large molecular mass of 1.25 mDa. Another VP8*-based vaccine candidate is the S_60_-VP8* pseudovirus nanoparticles that are composed of a T = 1 icosahedral norovirus inner shell with 60 surface-displayed VP8* antigens, having a large molecular weight of 1.47 mDa [35]. Although a direct comparison betewen these mentioned vaccine candidates has not yet been performed, the common immunological principle suggests that larger, polyvalent antigens, particularly those retaining more pathogen-associated molecular patterns (PAMPs), elicit higher immune responses compared with those induced by a smaller, single or low valent antigen. Thus, it should be logical to anticipate that the P_24_-VP8* and the S_60_-VP8* nanoparticles are more immunogenic than the P2-VP8* proteins. It is noted that the P2-VP8* vaccine with a T cell epitope may help to increase immune response at a certain level [55]. On the other hand, the P_24_-VP8* nanoparticle contains the P domain that is the receptor-binding protein of norovirus [56], offering a possible dual vaccine capability against both rotavirus and norovirus [15]. However, ultimate answers to these speculations will need further studies to address those issues directly.

Regarding the P_24_-VP8* nanoparticle-ligand binding function, we would like to clarify the following scenario. The norovirus P-domain-based P_24_ nanoparticle is known to bind HBGAs [17,43,57] that is a nature of norovirus for viral infection [56,58]. However, the insertion site of the VP8* antigens to the selected surface loop (loop 2) is a key component of norovirus HBGA binding site [59,60]; in other words, the chimeric P-VP8* protein destructs the HBGA binding function of norovirus P domain and thus the P_24_ nanoparticles. As a result, the observed HBGA-binding functions of the P_24_-VP8* nanoparticles must be contributed exclusively by the displayed rotavirus VP8* proteins. This was further proved by the observed glycan binding specificities that match the rotavirus VP8* proteins but not the norovirus P domains.

## 5. Conclusions

The outcomes of this study collectively demonstrated that the trivalent P_24_-VP8* nanoparticles displaying rotavirus VP8* of P[8], P[4], and P[6] types are a promising parenteral vaccine candidate for broad protection against rotavirus infection and diarrhea in both developing and developed worlds.

## Data Availability

Not applicable.
